# Observed and simulated submesoscale vertical pump of an anticyclonic eddy in the South China Sea

**DOI:** 10.1038/srep44011

**Published:** 2017-03-09

**Authors:** Yisen Zhong, Annalisa Bracco, Jiwei Tian, Jihai Dong, Wei Zhao, Zhiwei Zhang

**Affiliations:** 1 Institute of Oceanology, Shanghai Jiao Tong University, 800 Dongchuan Road, Shanghai 200240, P. R. China; 2 Earth and Atmospheric Sciences, Georgia Institute of Technology, 311 Ferst Drive, Atlanta 30332, USA; 3 Physical Oceanography Laboratory/Qingdao Collaborative Innovation Center of Marine Science and Technology, Ocean University of China, 238 Songling Road, Qingdao 266100, P.R. China; 4 Oceanic Modeling and Observation Laboratory, Marine Science College, Nanjing University of Information Science and Technology, 219 Ningliu Road, Nanjing, 210044, P.R. China

## Abstract

Oceanic mesoscale eddies with typical sizes of 30–200 km contain more than half of the kinetic energy of the ocean. With an average lifespan of several months, they are major contributors to the transport of heat, nutrients, plankton, dissolved oxygen and carbon in the ocean. Mesoscale eddies have been observed and studied over the past 50 years, nonetheless our understanding of the details of their structure remains incomplete due to lack of systematic high-resolution measurements. To bridge this gap, a survey of a mesoscale anticyclone was conducted in early 2014 in the South China Sea capturing its structure at submesoscale resolution. By modeling an anticyclone of comparable size and position at three horizontal resolutions the authors verify the resolution requirements for capturing the observed variability in dynamical quantities, and quantify the role of ageostrophic motions on the vertical transport associated with the anticyclone. Results indicate that different submesoscale processes contribute to the vertical transport depending on depth and distance from the eddy center, with frontogenesis playing a key role. Vertical transport by anticyclones cannot be reliably estimated by coarse-resolution or even mesoscale-resolving models, with important implications for global estimates of the eddy-driven vertical pumping of biophysical and chemical tracers.

Coherent vortices at the oceanic mesoscale (30–200 km), so called eddies, are ubiquitous in the world ocean and are key to transporting oceanic physical and biogeochemical tracers^1^,^2^,^3^,^4^. They stir them along isopycnals^5^, trap fluid at their interior, maintain for most of their lifespan the local properties of when and where they were formed^6^,^7^, and are major contributor to the spatial variability of the main pycnoclines creating domes and depressions through an eddy uplift mechanism, known in the literature as eddy pumping^8^,^9^. In a geostrophically balanced ocean, eddy pumping results primarily from the stress caused by the differences between surface winds and eddy velocities that induces Ekman upwelling, and shoaling of density surfaces, in the core of cyclones, and downwelling, with consequent deepening of the isopycnals, in anticyclones. Most eddies are surrounded by an annular vorticity cell at their edge, cyclonic around anticyclones and vice versa^10^. The eddy-induced Ekman pumping takes place also at the eddy periphery, where it can be stronger but of opposite sign than in the core^11^,^12^. The interaction of the surface stress with the vorticity gradient further generate a dipolar structure that modulates these upwelling and downwelling^13^,^14^. Estimates based on mesoscale-resolving observations and both realistic and idealized models suggest that the eddy uplift may account for as much as 20–30% of new production in subtropical gyres^1^,^15^,^16^,^17^,^18^.

Recently, attention has been in paid to the contribution of submesoscale circulations to the transport and mixing within and across the surface mixed-layer^19^,^20^,^21^. Here the term ‘submesoscale’ broadly applies to circulations characterized by a Richardson and a Rossby number of order O(1) and vertical velocities akin to those found in frontal systems (10–100 m d^−1^)^22^. Most research on this topic, however, concentrated on circulations such as submesoscale eddies and fronts and on their role in restratifying the surface mixed layer^23^,^24^,^25^.

Quantifying the contribution of submesoscale dynamics at the edge and within the core of eddies to the overall eddy transport remains an important but challenging problem, and is key to our understanding of biogeochemical interactions, primary productivity and export production^14^,^21^. The few modeling exercises that have concentrated on this issue suggest that upwelling and downwelling rates associated with submesoscale, ageostrophic circulations may be an order of magnitude larger than those estimated using the quasi-geostrophic omega equation^20^,^26^,^27^. Even fewer studies attempted to characterize from an observational standpoint the vertical velocity field inside mesoscale eddies, with noticeable exceptions being the survey of an anticyclone in the North Atlantic on a 2 × 20 km grid by Martin and Richards^28^ and the sampling of an anticyclone in the Algerian basin along its main axes at 4 km resolution using a glider by Cotroneo *et al*.^29^. Models and observations qualitatively agree in revealing intense vertical motions associated with alternating bands of positive and negative velocities, but the overall impact of those ageostrophic circulations on the time-averaged eddy vertical transport is uncertain^14^,^28^. Despite attempts to reconstruct distribution and mean structure of the eddy field in various ocean basins with composite analyses of satellite altimeter and *in-situ* data^13^,^30^,^31^, our knowledge of the 3D structure of oceanic eddies remains incomplete.

Here for the first time we explore the submesoscale structure of an anticyclone using *in-situ* data from a high-resolution, submesoscale permitting survey conducted in the winter of 2014 in the South China Sea (SCS), a basin populated by energetic mesoscale eddies as revealed by both observational and modeling studies^32^,^33^,^34^,^35^,^36^. The winter sampling, at a time of maximum mixed-layer depth, allows for capturing submesoscale circulations at their maximum strength^37^,^38^,^39^. The observed eddy structure is then compared to that simulated in three integrations, one at 10 km (MP for mesoscale permitting), one at 5 km (MR, for mesoscale resolving) and one at 1 km (SP, for submesoscale permitting) horizontal resolution. MP is close to the target of the high resolution coupled climate models that will participate in the next intercomparison project (CMIP6). Our goals are to establish which resolution is required to capture the observed statistics inside the eddy, and evaluate the error introduced by using coarser models. Once the resolution requirement is established, the vertical transport within the eddy is quantified in the model using three-dimensional Lagrangian particles. Such comparison highlights the contribution of ageostrophic, small-scale circulations to the vertical transport within the eddy and its periphery while explaining the mechanisms responsible for patterns of upwelling/downwelling inside anticyclones at different water depths.

## Results

### Observations

**Figure 1:**
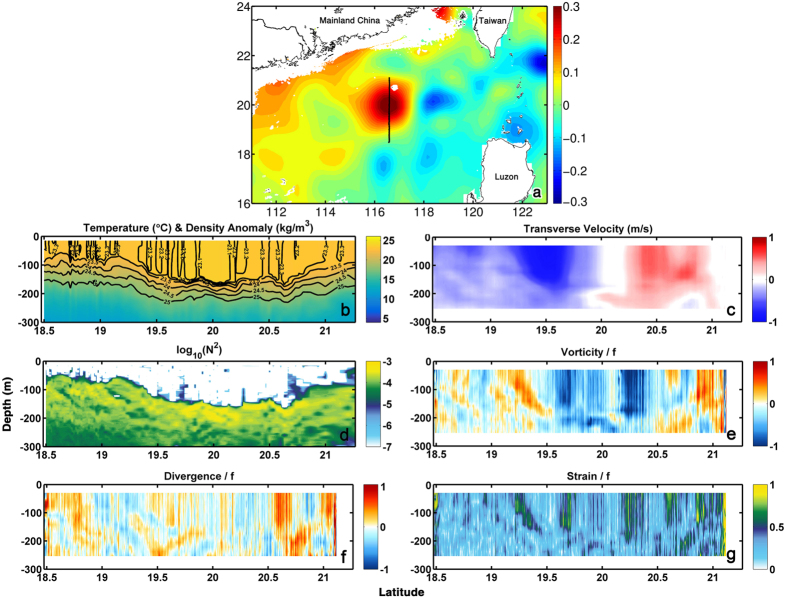
(**a**) Sea surface height anomaly in correspondence of the observed mesoscale eddy from AVISO satellite altimeter data (http://www.aviso.altimetry.fr). The black line indicates the transect along which measurements were taken. (**b**) Temperature, (**c**) Transverse velocity, (**d**) Buoyancy frequency, (**e**) Relative vorticity, (**f**) Horizontal divergence and (**g**) Strain along the transect in panel (**a**). The figure was made using MATLAB R2010b (http://www.mathworks.com).

During Jan 14–16, 2014, the R/V Dongfanghong 2 conducted a high-resolution survey of the northern South China Sea (SCS) as part of an effort to understand its multi-scale dynamics. The survey focused on a large anticyclonic eddy, about 200 km in diameter, and the measurement was carried out approximately along 116.5°E (+/−0.01°) from 18.5°N to 21°N (Fig. 1a). The generation and dissipation processes of the same anticyclone have been investigated through a mooring array deployed in early 2013 and recovered in June 2014, revealing a deep-reaching 3-D barotropic structure with a tilted vertical axis extending to the ocean bottom^36^. The anticyclone was generated by an intrusion of the Kuroshio Current into the SCS and subsequent eddy shedding.

Current velocities were measured with an Acoustic Doppler Current Profiler (ADCP), and hydrographic quantities with a Conductivity-Temperature-Depth (CTD), a microstructure MSS90 profiler and a number of Expendable Bathythermographs (XBT) at submesoscale-resolving sampling intervals (see Methods). Our high-resolution observations extend to 300 m for the CTD casts and 250 m for the ADCP data, capturing in detail the upper portion of the eddy core as shown in the potential temperature and density profiles (Fig. 1b). The survey followed an eddy diameter, measuring a zonal/circumferential flow in excess of 1 m s^−1^ in its core (Fig. 1c) and a meridional/radial flow about one order of magnitude smaller (not shown). The buoyancy frequency along the transect 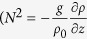, where *g* is the gravitational acceleration, *ρ* is potential density and *ρ*_0_ a reference density) ranged between 10^−4^ and 10^−3^ s^−2^ in correspondence of the pycnocline (Fig. 1d). Values three order of magnitude lower characterized the mixed-layer everywhere but for narrows spikes both inside the eddy and near its periphery where *N*^2^ reached 10^−5^ s^−2^. Despite this slightly enhancement in *N*^2^ associated with greater lateral density gradients, no remarkable restratification was found since there is a distinct upper mixed layer both inside and outside the eddy^40^,^41^.

Figure 1e–g show also relative vorticity, horizontal divergence and strain normalized by the Coriolis parameter (see Methods). Bands of positive relative vorticity values around or above one are attained at the outer edges of the core of the anticyclone, and negative values as small as −0.8 characterize the eddy interior. Strain is enhanced at the same locations as the vorticity extrema. The normalized horizontal divergence field shows no simple correlation with vorticity, and is indicative of strong ageostrophic secondary circulations^24^,^25^ and elevated vertical velocities. Large divergence values are found at the edge of the eddy in agreement with numerical predictions^19^,^20^, but values as large as 0.5 are attained also in the anticyclone interior. Our observations capture for the first time the intensity and spatial variability of the ageostrophic submesoscale circulation within an eddy and represent an important step towards quantifying the contributions of anticyclonic eddies to vertical transport from the ocean surface to below the mixed layer.

### Modeling: Eulerian fields

The objective of the modeling work is to establish the resolution requirements to capture the statistics of the observed dynamical quantities, to investigate the submesoscale processes at play in anticyclonic eddies and to quantify the role of the vertical fluxes associated to the representation of the submesoscale circulations. Eddies such as that observed in January 2014 are commonly found in the northern SCS, where they periodically form from the interaction of the flow entering the basin from the Pacific Ocean and the SCS bathymetry, but are also generic representative of the mesoscale anticyclones that populate other tropical and subtropical regions^13^.

**Figure 2:**
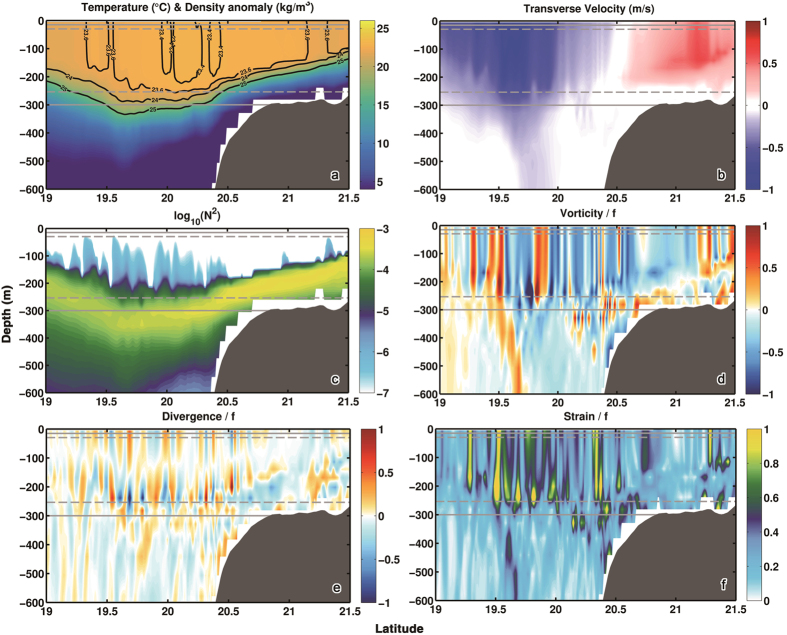
Temperature (**a**), Transverse velocity (**b**), Buoyancy frequency (**c**), Relative vorticity (**d**), Horizontal divergence (**e**) and Strain (**f**) along 116.5°E transect in the SP run. The figure was made using MATLAB R2010b (http://www.mathworks.com).

**Figure 3:**
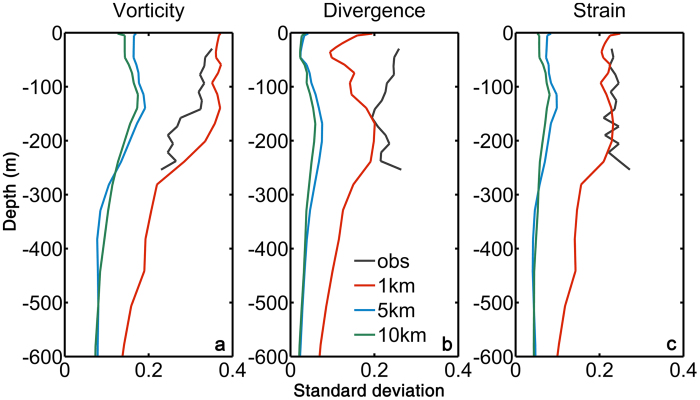
Vertical profiles of standard deviation of: Relative vorticity (**a**), Horizontal divergence (**b**) and Strain (**c**). The figure was made using MATLAB R2010b (http://www.mathworks.com).

To make our conclusions as general as possible, the circulation model is run without any data assimilation and through a different period than the cruise, from January 2000 to December 2002. We focus on an eddy formed in January 2001. Corresponding variables to those in Fig. 1b–g are presented along a transect at approximately the same longitude as in the observations (116.5°E) in the SP run in Fig. 2 and in the MR and MP simulations in Figs SI1 and SI2. The representation of the temperature field across the eddy is to a large degree independent of resolution. Submesoscale circulations affect mainly the horizontal density structure in the mixed layer but moderately the representation of the pycnoclines of the simulated eddy and the stratification in its interior, with smoother fields in MP and MR, as to be expected. Resolving submesoscale dynamics, however, results in far greater and structurally more complex vorticity, strain and horizontal velocity divergence fields. In Fig. 3 we verify that the modeled representation of the dynamical quantities in SP is in good agreement with those observed both in the interior and at the edges of the eddy by calculating the variance of vorticity, horizontal velocity divergence and strain. Only the horizontal velocity divergence appears to be slightly underestimated in the upper 120 m of the mixed layer. The mesoscale resolving or permitting simulations, on the other hand, underestimate the variance in each of the three fields considered by 3 to 5 fold.

### The vertical velocity field

Next we focus on the processes responsible for the complex dynamics in the anticyclonic eddy, using the observations and the SP experiment. Following McWilliams^42^, we consider three classes of instabilities: (1) gravitational instability, that may occur whenever the buoyancy frequency *N*^2^ changes sign; (2) symmetric instability or SI, for which a change in sign of Ertel potential vorticity (EPV) is a necessary condition^27^,^43^,^44^; and (3) ageostrophic anticyclonic instability (AAI), requiring a change in sign of (*A*–*S*) where *A* is absolute vorticity defined as *A* = *f* + *ζ* and *S* is strain rate 
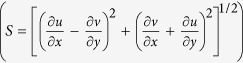
 42,45,46. An anticyclone is generally conductive to both AAI and SI, with the SI criterion being easily satisfied in the mixed layer where the stratification is weak and lateral density gradient can be strong.

**Figure 4:**
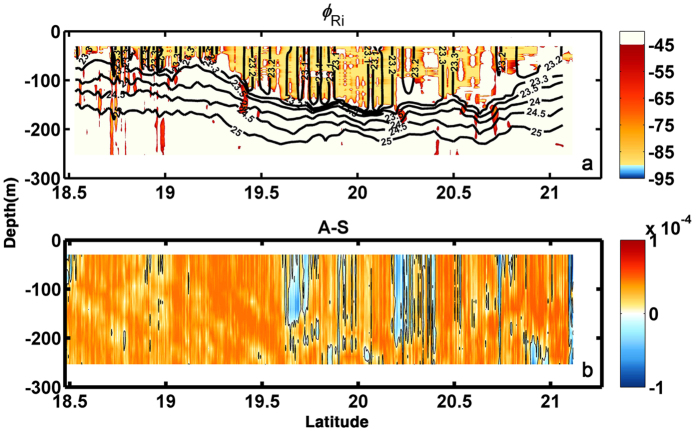
Vertical transect of the angle *ϕ*_Ri_ superposed with density contours (**a**) and vertical transect of *A*-*S* superposed with its zero-crossings (**b**). The figure was made using MATLAB R2010b (http://www.mathworks.com).

**Figure 5:**
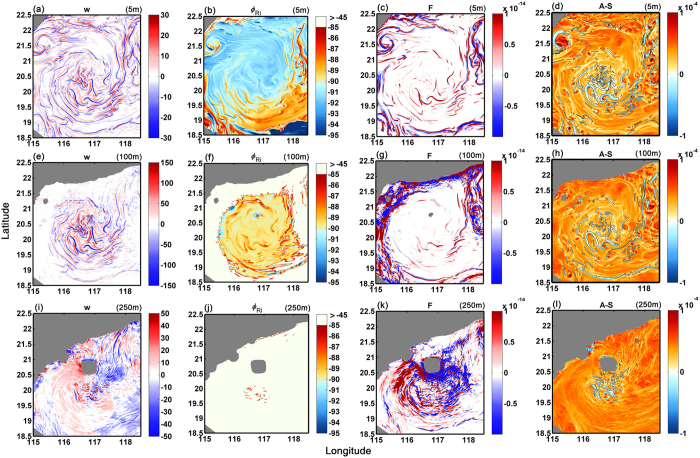
Vertical velocity (**a**,**e**,**i**, unit: m day^−1^), *ϕ*_Ri_ (**b**,**f**,**j**, unit: degree), frontal tendency (**c**,**g**,**k**, unit: kg^2^ m^−8^ s^−1^) and *A*-*S* (**d**,**h**,**l**, unit: s^−1^) of the SP simulations at 5 m (top), 100 m (middle) and 250 m (bottom). The contours on the *A*-*S* map are their zero-crossing lines. The figure was made using MATLAB R2010b (http://www.mathworks.com).

The instability criteria are verified in Fig. 4 for the observations along the transect and in Fig. 5 for the model data using plan views at three depth levels to highlight their spatial complexity. More specifically Fig. 4 shows the angle *ϕ*_Ri,_ introduced by Thomas *et al*.^44^ to differentiate between instability regimes (see Methods for details) and the observed (*A*-*S*). For an anticyclone values of *ϕ*_Ri_ comprised between −90° and −45° are indicative of conditions favorable to SI, while angles between −135° and −90° support a mixed symmetric/gravitational instability. Given that *in-situ* measurements near the surface are often contaminated by the ship track, we exclude the upper 15 m from our analysis. Notwithstanding a likely underestimation of the density gradients due to the CTD spatial sampling interval, *ϕ*_Ri_ is found to be in the range favorable to SI only in the mixed layer (Fig. 4a). The enhanced vertical stratification at the base of the mixed layer of the eddy inhibits the formation of SI favorable conditions in the pycnocline except for few patches when the tilting of the isopycnals is large enough to potentially exceed the slope of the isolines of constant absolute momentum.

Figure 4b shows (*A*-*S*) highlighting the zero-crossings. (*A*-*S*) remains largely positive over the whole transect with sharp sign changes in narrow areas mostly within the eddy core, where *A*/*f* < 1. These areas often extend through the mixed layer and penetrate into the pycnocline, where they may contribute to enhance diapycnal transport.

In Fig. 5a–l corresponding variables as well as vertical velocity *w* and frontal tendency *F (F* = ***Q*** · ∇_*h*_*ρ*, with 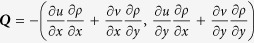)^25^ are plotted for the SP model output near the surface, in the bulk of the mixed layer at 100 m depth, and at the bottom of it, at 250 m. Near the ocean surface conditions are favorable to both symmetric and gravitational instability (*N*^2^ ~ −10^−8^) due to strong surface cooling characteristic of the winter season (Fig. 5b). SI conditions are most favorable to the south of the eddy, where northeastern winds and the consequent Ekman transport of adjacent warm water from west of Luzon^34^ help recover local gravitational stability. Conditions conductive to SI are also found at the same locations as positive frontogenetic tendency, because the frontogenetic strain can both steepen the isopycnals by increasing lateral density gradients and flatten the isosurfaces of the absolute momentum by increasing the vertical shear (Fig. 5c). At 100 m depth the whole eddy is symmetrically unstable with the *ϕ*_Ri_ being about −90° nearly everywhere (Fig. 5f), consistent with results from three-dimensional large eddy simulations^47^. Indeed at this depth both vertical stratification and lateral density gradients are weak. Conditions conductive to stronger SI are still associated with positive frontogenetic tendency organized in spiraling patterns (Fig. 5c,g), but *F* is weaker than at the surface particularly inside the eddy, suggesting that frontogenetic strain may suppress symmetric instability if too strong^48^. At the base of the mixed layer, only scattered symmetrically unstable regions can be seen in the eddy center, consistent with the observations (Fig. 5j). By comparing the pattern of SI, positive frontogenetic tendency and vertical velocity, we conclude that frontogenesis is the submesoscale process responsible for the pattern and strength of *w* at the surface, while SI becomes important in the middle of the mixed layer where it can develop in correspondence of the weaker fronts.

Focusing on (*A*-*S*), the zero-crossings coincide broadly with strong vertical velocities at all depth, particularly at the center of the eddy core, where high *w* values cannot be explained by frontogenesis or SI (Fig. 5d,h,l). This suggests that AAI plays a significant role in setting up the submesoscale vertical velocity structure in the center of anticyclonic eddies. A wave-like pattern is also found in the vertical velocity and frontogenesis map near the bathymetry at 250 m (Fig. 5i,k), likely due to the lee waves induced by the interaction of the anticyclonic flow and a sea mountain.

Fields equivalent to those shown in Fig. 5 are plotted for the MR and MP integrations in Figs SI3 and SI4, respectively. Vertical velocity and frontal tendency are much weaker and less complex at lower resolution. SI favorable conditions are found in both runs consistent with the SP integration but constrained to the mixed layer only, while the criterion for AAI is never satisfied. This further supports that the complex submesoscale circulations seen in the observations and in the high resolution model are a consequence of concurrent strong frontogenesis and AAI.

Finally, we briefly describe the frequency spectra of the vertical velocity field inside the eddy (see Methods), being relevant to interpreting the transport analysis. In anticyclones the local or effective planetary vorticity is for most part reduced being *f*_*eff*_ = *f* + *ζ*/2 and ζ negative, leading to a trapping of internal waves of near-inertial frequency (NIWs) at their interior and downward energy propagation^49^,^50^. In SP the higher vertical velocities in the mixed layer reflect the contribution of all frequencies, with the superinertial increasing the most for increasing resolution (Fig. SI5). Recent work described the mechanism by which NIWs modulate the strength of superinertial internal waves (SSIW) inducing a substantial increase in SSIW energy in convergence zones, resolved only in SP^51^. Below 100 m depth, the increase in energy per increasing resolution is evident at all frequencies but the near inertial peak becomes dominant, as predicted theoretically^52^ and discussed in previous works^20^,^26^. Consequently below the mixed layer NIWs dominate the *w* field and impact more effectively the transport.

### Modeling: Eddy transport

Under the assumption that a model capable of reproducing variance and mean of horizontal velocity divergence, strain and vorticity is also providing a reliable representation of the vertical velocity field, we evaluate how the representation of vertical transport in the anticyclone varies with resolution by releasing neutrally-buoyant Lagrangian tracers freely to move in all three directions in each simulation at the time shown in Fig. 5. Particles are released over the eddy and its circulation cell near the surface (5 m), at the approximate depth of the deep chlorophyll maximum, about 50 m^53^, and at the base of the mixed layer outside the eddy perimeter, at about 100 m depth (see Methods for details).

**Figure 6:**
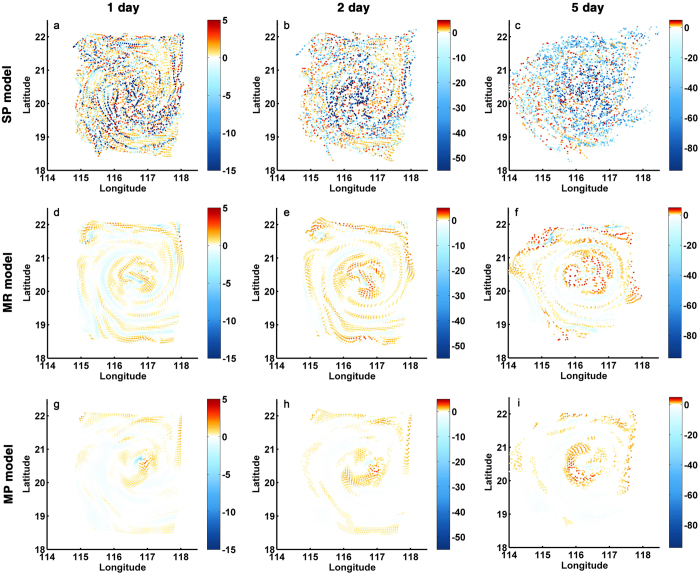
Three-dimensional distribution of particles initially deployed at 5 m after 1 day (**a**,**d**,**g**), 2 days (**b**,**e**,**h**) and 5 days (**c**,**f**,**i**) in the SP (top), MR (middle) and MP (bottom) simulations. Color shading indicates the particle displacement, positive for upward and negative for downward. The figure was made using MATLAB R2010b (http://www.mathworks.com).

**Figure 7:**
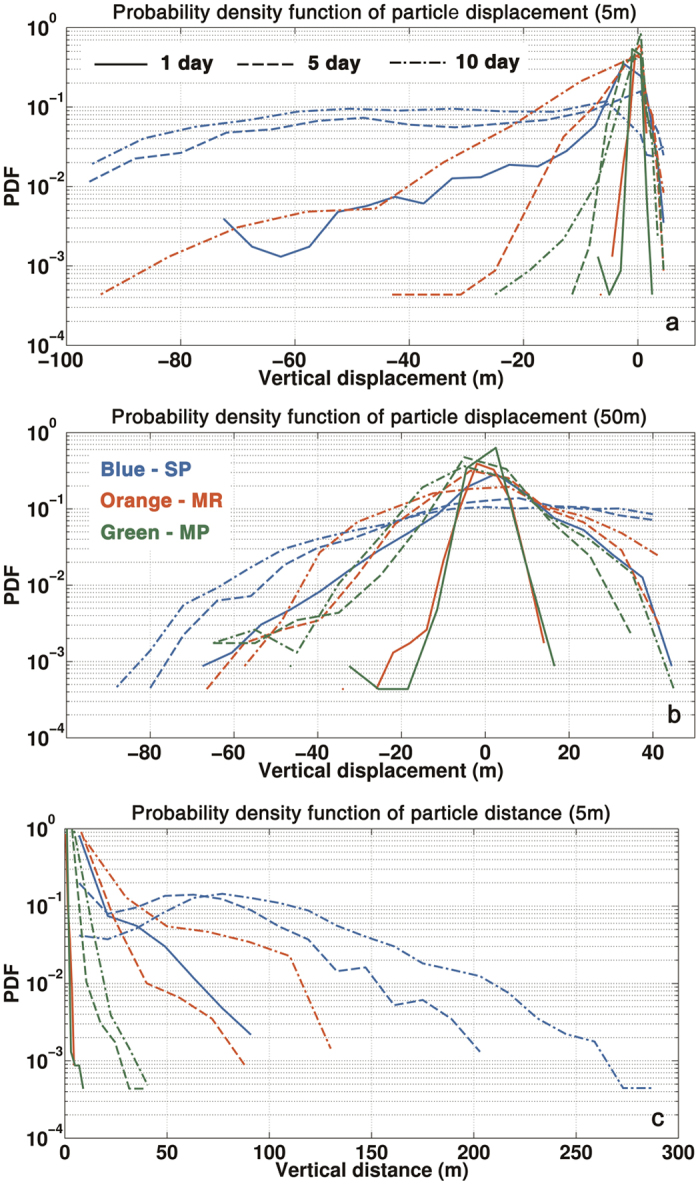
(**a**) Probability density function of the total vertical displacement traveled by the particles initially deployed at 5 m after 1 day (solid), 5 days (dash), 10 days (dash-dot) in the SP (blue), MR (red) and MP (green) simulations; (**b**) Same as (**a**) but for the particles initially deployed at 50 m; (**c**) Same as (**a**) but for the PDF of the total vertical distance covered by the particles initially deployed at 5 m. The figure was made using MATLAB R2010b (http://www.mathworks.com).

The three-dimensional evolution of the particles released at the surface is shown in Fig. 6. In the SP case the tracers undergo a vigorous downward vertical motion in correspondence of the spiraling frontal features in Fig. 5, with nearly 11% of them sinking below 20 m within the first day, 25% by the end of the second day, and more than 51% doing so within five days, as quantified in Fig. 7. In the MR case the vertical displacement of the ensemble of particles is also characterized by a spiraling pattern but is limited in amplitude, with the majority (87%) of particles remaining within the upper 10 m of the water column during the first five days. The MP simulation is characterized by a vertical displacement analogous to the MR run in the core of the eddy but further reduced at the eddy periphery. Vertical transport is enhanced whenever submesoscale circulations are accounted also for the 50 m release (Fig. SI6) and particles in the SP simulation travel more efficiently both upward, mostly at the eddy periphery, and downward, preferentially in the eddy core. After five days a significant number of tracers in SP have reached depths greater than the region of influence of the submesoscale fronts. In the MR simulation, on the other hand, particles remain for longer within shallower depths where the weaker and wider spiraling patterns dominate the vertical transport pattern (Fig. SI6). In the release at 100 depth differences between the SP, MR and MP simulations remain especially evident at the eddy core where AAI continue playing an important role if resolved (Fig. SI7).

Figure 7 quantifies the model resolution dependency in the representation of vertical transport by displaying the histogram of the particle vertical displacement around the deployment depth after 1, 5 and 10 days for 5 m and 50 m releases, and the overall vertical distance covered by the near-surface tracers after the same time intervals. Particles released near the surface are found in nearly identical concentrations at depths comprised between 10 and 70 m after 5 or 10 days in the SP run, while they remain concentrated near the surface in the 5 km resolution case. No particles reach depths greater than 25 m in the MP run. For deployments at 50 and 100 m depth, doubling horizontal resolution from 10 to 5 km does not modify substantially the transport representation. Both upward and downward motions are significantly underestimated in the 50 m deployment case if submesoscale vertical velocities are not accounted (Fig. 7b).

**Figure 8:**
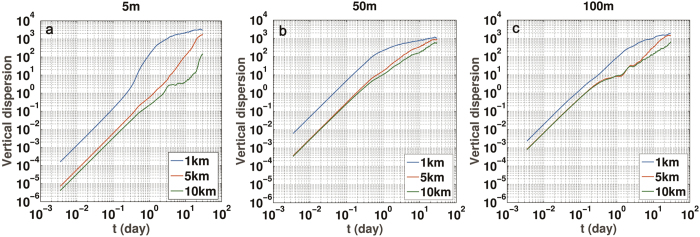
Vertical dispersion of the particles initially at 5 m (**a**), 50 m (**b**) and 100 m (**c**) in the SP (blue), MR (red) and MP (green) simulations. The figure was made using MATLAB R2010b (http://www.mathworks.com).

Vertical absolute dispersion quantifies the transport differences at 5 m, 50 m and 100 m depths in Fig. 8. It is over one order larger in the SP case than in MR or MP for particle released at 5 and 50 m over the first 3–5 days, with differences reduced to about half after that. Initially all dispersion curves follow a ballistic regime lasting between 4 hours and 12 hours^20^,^54^,^55^. In the case of particles deployed near the ocean surface, the SP and MR or MP curves diverge even further at intermediate times due to the large discrepancies in the representation of vertical velocities between the runs. From day 4 in SP and day 20 in MR to the end of the integration particles follow a slightly sub-diffusive regime, with *A*_z_^2^(*t*) ~ *t*^0.5–0.75^. This regime becomes prominent once enough particles have reached depths greater than 150 m where the near-inertial motions dominate the energy spectrum of the vertical velocity field causing alternating positive and negative displacements, and is achieved earlier by the tracers released at 100 m and last by those initially at the surface, and first in SP and then in the MR and MP runs.

Overall our analysis indicates that submesoscale circulations are very effective contributors to the vertical transport of nutrients, carbon and oxygen inside anticyclonic eddies both downward, away from the mixed layer, and upward, across the base of the euphotic layer.

## Discussion

Eddies are key to the three-dimensional transport of physical and biological tracers in the ocean. Along the vertical direction, eddy Ekman pumping is linked, in a geostrophically balanced view, to the raising of the pycnocline, and therefore upwelling, in cyclonic cores, and to its depression, with downwelling motions, in anticyclones. Recently, however, limited measurements and modeling investigations have raised the possibility for ageostrophic circulations to impact significantly upwelling and downwelling rates in the core and periphery of ocean eddies^14^,^21^,^26^,^28^,^56^.

Here, we present observational evidence for strong ageostrophic motions in an anticyclonic eddy extending to the upper 300 m of the water column through tightly spaced observations in the northern South China Sea. The flow derived from ADCP measurements is characterized by patches with relative vorticity normalized by the Coriolis parameter – and therefore by a local Rossby number - of order one, with elevated values of both strain and horizontal velocity divergence, and is consistent with the simulation of the surveyed region by a submesoscale permitting model run at 1 km horizontal resolution. While 1 km may not be sufficient to fully capture the complexity of the observed fields, and indeed the variance of the horizontal velocity divergence field is slightly underestimated, it represents a good compromise between including ageostrophic, submesoscale dynamics and avoiding spurious overestimations of the vertical velocity field near continental shelves and ocean ridges, where solitary waves and their dispersion are controlled by nonhydrostatic processes^20^,^57^.

An analysis of the modeled patterns reveals that mean and variance in horizontal velocity divergence and vorticity observed at the transect are consistent with the presence of ageostrophic fronts where conditions favorable to symmetric instability are also encountered. The ageostrophic vertical velocities enhance transport across the upper water column not only in the circulation cell that delineates the outer edge of the eddy, but also in the eddy and its core. The impact of such enhancement on vertical transport has been evaluated comparing the evolution of particles released in the anticyclone near the ocean surface, at 50 m and at 100 m in three simulations at 1, 5, and 10 km horizontal resolution. Transport, quantified here by absolute dispersion, is three to five times greater in the submesoscale permitting case after one month and by over an order of magnitude over periods of one to ten days. For particles released at 100 m differences are partially attenuated due to an overall cancellation of the upwelling and downwelling contributions mostly associated with the near-inertial field.

The results presented confirm the fundamental role played by submesoscale ageostrophic dynamics in determining vertical transport in the upper water column not only in frontal regions or whenever submesoscale eddies are abundant^20^, but also in mesoscale eddies. In the case of the sampled anticyclone submesoscale circulations affect dynamical quantities in the core as much as in the circulation cell. Although the anticyclone has characteristics that are typical of the eddy population of the northern South China Sea^36^, its upper structure is generic. Indeed Loop Eddies in the Gulf of Mexico simulated at submesoscale permitting resolution confirm the presence of spiraling regions of elevated vertical velocity and horizontal convergence during winter and early spring^58^. We verified that in those simulations the variance of vorticity, horizontal divergence and strain, and transport properties in the Loop Eddies are indistinguishable from those observed and modeled in the South China Sea.

Based on the new observational evidence and on our modeling results, we conclude that in winter the vertical transport due to mesoscale anticyclones, both upward and downward within the mixed layer, is at least one order of magnitude greater than estimated through state-of-art, mesoscale resolving models. Resolution will need to increase by a factor of ten before resolving explicitly submesoscale circulations and accounting for their role on vertical transport of heat and tracers in the ocean. Current submesoscale parameterizations consider the impact of submesoscale circulations on vertical transport in relation to their restratifying contribution^59^,^60^,^61^. Our observational and modeling analysis indicates that submesoscale fronts in and around anticyclones are key contributors to the vertical velocity field and to vertical transport of the overall eddy structure without any detectable restratification.

## Methods

### *In-situ* measurement

The velocity data were obtained from a shipboard Teledyne RDI 75 kHz Acoustic Doppler Current Profiler (ADCP), transmitting in 16 m vertical bins every 1′, or equivalently at a horizontal resolution of 110 m. The average ship speed was around 4.5 m/s. Data was then averaged over 500 m horizontal bins to reduce the noise level while still resolving submesoscale dynamics. CTD (Seabird 911 Conductivity, Temperature, Depth) and MSS90 (Sea & Sun Microstructure Profiler) profiles were taken alternatingly to depths of about 300 m at intervals varying from 3′ to 4′ and all data points were aligned to the 116.5°E line, roughly along the eddy north-south diameter. To test the consistency between these two instruments, they were deployed together at three stations (19°N, 20°N and 21°N). Temperature profiles agreed well but the salinity measured by MSS90 was generally 0.06 PSU larger than that measured by the CTD over the whole water column. MSS90 salinities were corrected by shifting them towards the CTD values using least square calculations. Between CTD and MSS90 casts, approximately every 2 km an Expendable Bathythermograph (XBT) was dropped in the sea to measure the temperature over the whole water column. Density from CTD and MSS90 data was calculated using the Gibbs Seawater (GSW) Oceanographic toolbox^62^. The results are used as a training dataset to determine the temperature-density relationship in a quadratic form of least square fitting. We derived the density profiles associated with the XBT data based on this formula.

### Estimates of derived variables from ADCP data

Scaled vertical component of relative vorticity, divergence, and strain were calculated in cylindrical coordinates (*r*, *θ*) as:


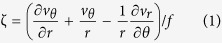



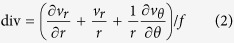



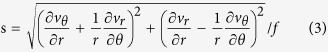


where *v*_*r*_ and *v*_*θ*_ are the radial and the azimuthal velocities and *f* is planetary vorticity.

In evaluating these variables along the eddy diameter, we neglected the azimuthal variation terms ∂/∂*θ* by assuming a symmetric structure of the eddy. This assumption is generally acceptable except near the eddy center, where the divergence is significantly overestimated due to a large curvature term 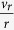. Within 5 km from the eddy center we simply omitted this term as well and retained only the ∂/∂*r* terms. Using model data we verified that the patterns associated with the above simplification remain similar, though the magnitude is slightly higher, particularly in the divergence field in the center of the eddy where the overestimation can reach 10% at few points (Fig. SI8).

### Numerical model set-up

We performed all simulations using the Regional Ocean Modeling System (ROMS) in the IRD (Institute of Research for Development) version of the code, aka ROMS-AGRIF^63^,^64^. ROMS is a three-dimensional, free surface, hydrostatic, primitive-equation circulation model with a generalized terrain-following coordinate^65^. We adopted the split third-order upwind scheme with rotated diffusion operator for horizontal tracer advection to reduce spurious diapycnal mixing^66^, harmonic viscosity with coefficient 5 m^2^/s, linear bottom drag with coefficient 3 × 10^−4^ m/s and the nonlinear K-Profile Parameterization (KPP) for vertical mixing of tracers and momentum at the surface and bottom boundary layers^67^.

Simulations were configured over the whole SCS (approximately 100°E–130°E, 3°N–32°N) for the 10 km and 5 km horizontal resolution cases, with 32 sigma layers in the vertical, of which 13 confined within the upper 150 m of the water column. The model was spun up for 20 years with climatological monthly-varying forcings (1948–2002 NCEP monthly climatologies)^68^ to reach a statistically stationary state and then integrated over 2000–2008 with realistic forcings (6-hourly QSCAT/NCEP blended satellite wind^69^ and 6-hourly NCEP heat and fresh water fluxes^68^). The Simple Ocean Data Assimilation version 2.1.6 (SODA)^70^ provided initial and boundary conditions. The 5 km grid was then refined to 1 km in the northern part of the basin (113°E–122°E, 17°N–25°N) using offline nesting^71^ and run again from November 1, 2000 to the end of March, 2001. 3-day averaged output from the 5 km run provided the initial and boundary conditions. All the parameterization and numerical schemes were unchanged.

### Angle

In evaluating the instabilities responsible for submesoscale circulations in the eddy we followed the work by Thomas *et al*.^44^ in which the use of the variable ***ϕ***_**Ri**_ defined as


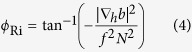


is proposed.

For anticyclonic vorticity, gravitational instability can occur whenever *ϕ*_Ri_ is between −180° and −135°; mixed symmetric/gravitational instability for −135° < *ϕ*_Ri_ < −90°; pure symmetric instability whenever *ϕ*_Ri_ is between −90° and −45°; mixed symmetric/inertial instability for *ϕ*_Ri_ between −45° and *ϕ*_c_; no instability if *ϕ*_Ri_ is between *ϕ*_c_ and 0°. Here *ϕ*_c_ is a critical value defined as 
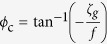
, where *ζ*_*g*_ is geostrophic vorticity.

### Estimating frequency spectra of vertical velocity

The modeled vertical velocity fields were saved every hour over a 30-day period. The frequency spectra were estimated for each spatial point using the periodogram method applied to the hourly data. Figure SI5 shows the ensemble mean spectra for different depth levels.

### Lagrangian particles and Vertical dispersion

A ROMS offline tracking module was used to calculate the Lagrangian trajectories from stored ROMS velocity fields^72^. The neutrally buoyant Lagrangian particles are free to move in all three directions and are advected forward in time by the hourly average Eulerian velocity field 
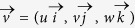
 linearly interpolated at the particle location 
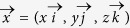
 according to the equation.


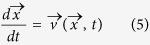


4331 particles were released over the eddy and its circulation cell (115°E–118°E, 18.5°N–22°N) at each depth level of the integration independently of resolution.

No additional vertical diffusion term was added to the particles, so that our estimates of vertical transport represent a lower bound. Horizontal diffusion was accounted by the parameterization of harmonic mixing. The horizontal velocity field responsible for the horizontal dispersal of the Lagrangian particles results indeed from the sum of an advective term and a harmonic viscous term.

Vertical dispersion was calculated from the Lagrangian tracers’ position as


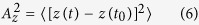


where *z(t*) is the vertical displacement of a particle at time *t*, *t*_0_the release time and the angle brackets indicate ensemble mean.

## Additional Information

**How to cite this article:** Zhong, Y. *et al*. Observed and simulated submesoscale vertical pump of an anticyclonic eddy in the South China Sea. *Sci. Rep.* **7**, 44011; doi: 10.1038/srep44011 (2017).

**Publisher's note:** Springer Nature remains neutral with regard to jurisdictional claims in published maps and institutional affiliations.

## Supplementary Material

Supplementary Figures
