# Supplementary material for: Observed and simulated submesoscale vertical pump of an anticyclonic eddy in the South China Sea
Source: Sci Rep. 2017 Mar 9;7:44011. doi: 10.1038/srep44011 (PMC5343664; doi:10.1038/srep44011)
Supplement: Supplementary Figures [file srep44011-s1.pdf]

Observed and simulated submesoscale vertical pump of an anticyclonic eddy in the South China Sea

Yisen Zhong[1], Annalisa Bracco[2], Jiwei Tian[3]*, Jihai Dong[3], Wei Zhao[3], Zhiwei Zhang[3]

[1] Institute of Oceanology, Shanghai Jiao Tong University, 800 Dongchuan Road, Shanghai 200240, P. R. China.
[2] Earth and Atmospheric Sciences, Georgia Institute of Technology, 311 Ferst Drive, Atlanta 30332, USA.
[3]Physical Oceanography Laboratory/Qingdao Collaborative Innovation Center of Marine Science and Technology, Ocean University of China, 238 Songling Road, Qingdao 266100, P.R. China.
* Correspondence and requests for materials should be addressed to J.T. (email: tianjw@ouc.edu.cn)

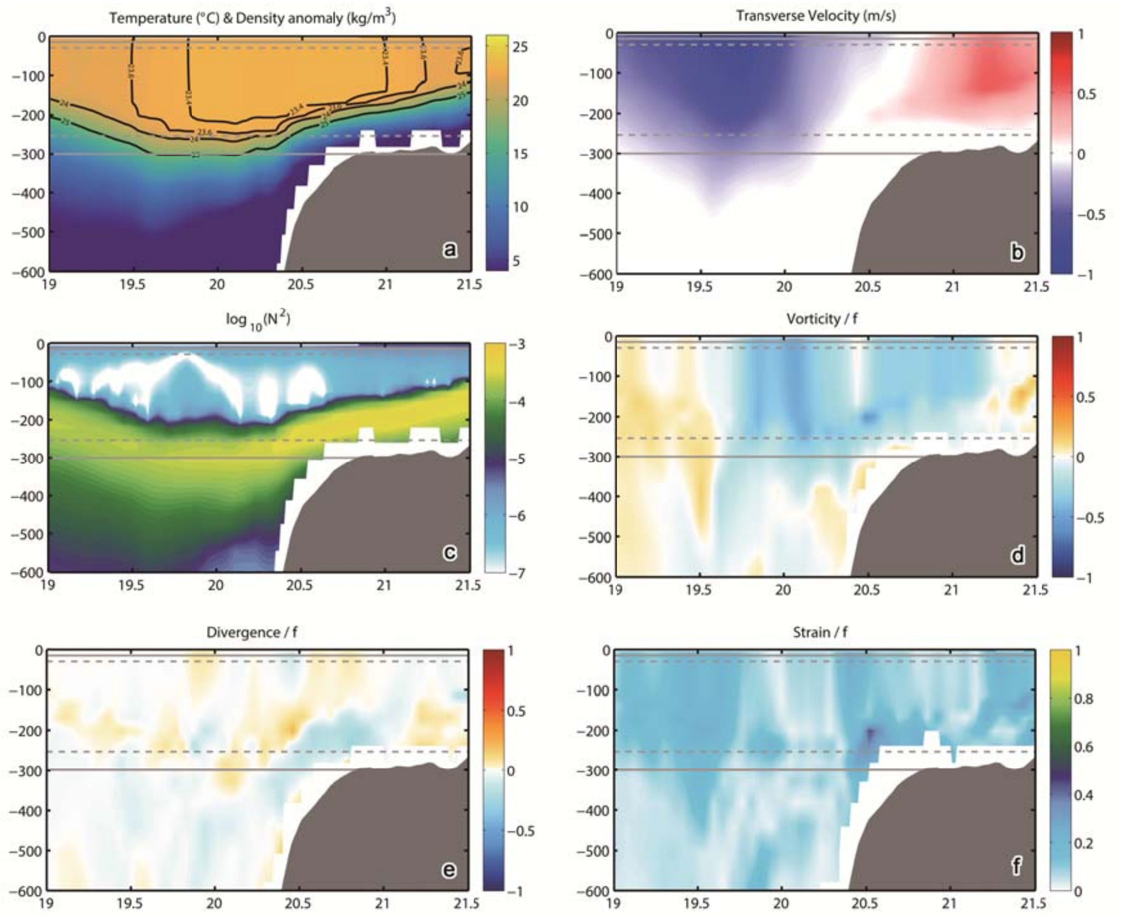

Figure SI 1: Temperature (a), Transverse velocity (b), Buoyancy frequency (c), Relative vorticity (d), Horizontal divergence (e) and Strain (f) along 116.5°E transect in the MR run. The figure was made using MATLAB R2010b (http://www.mathworks.com).

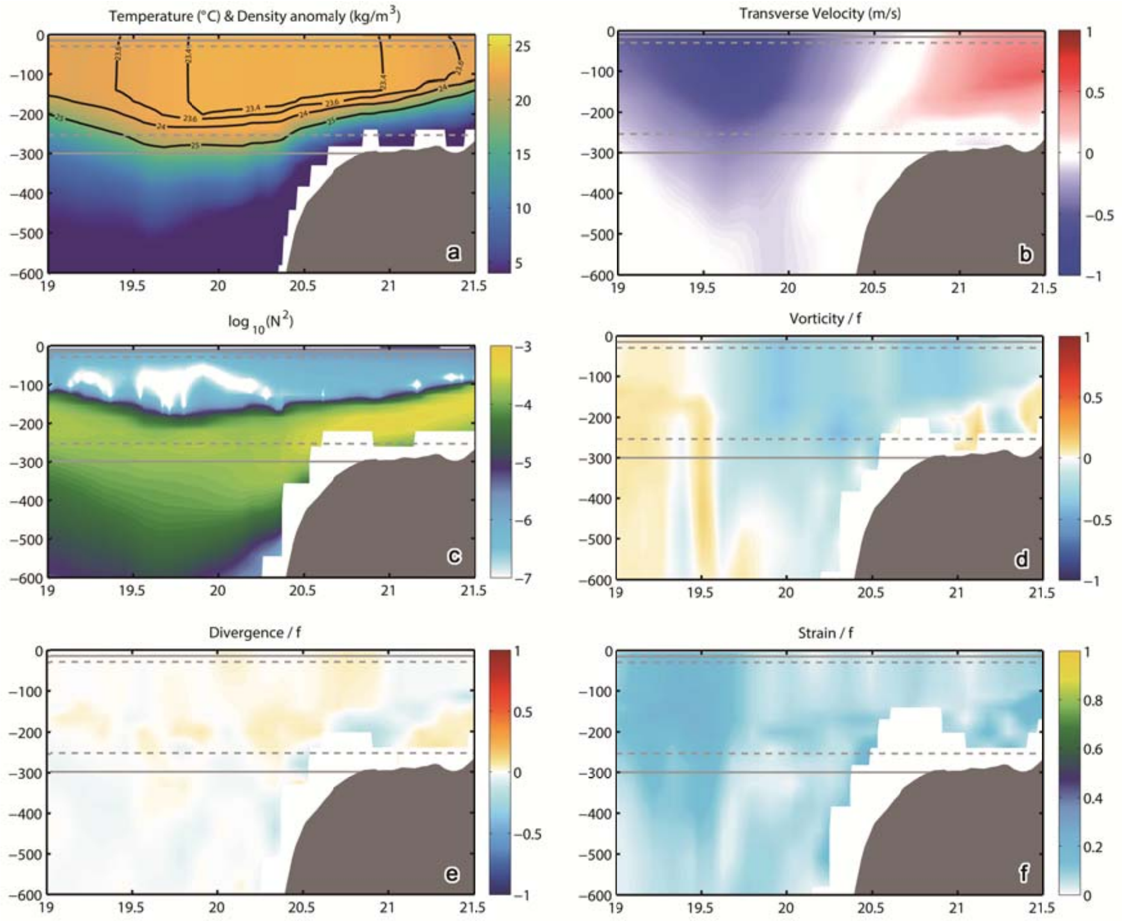

Figure SI 2: Temperature (a), Transverse velocity (b), Buoyancy frequency (c), Relative vorticity (d), Horizontal divergence (e) and Strain (f) along 116.5°E transect in the MP run. The figure was made using MATLAB R2010b (http://www.mathworks.com).

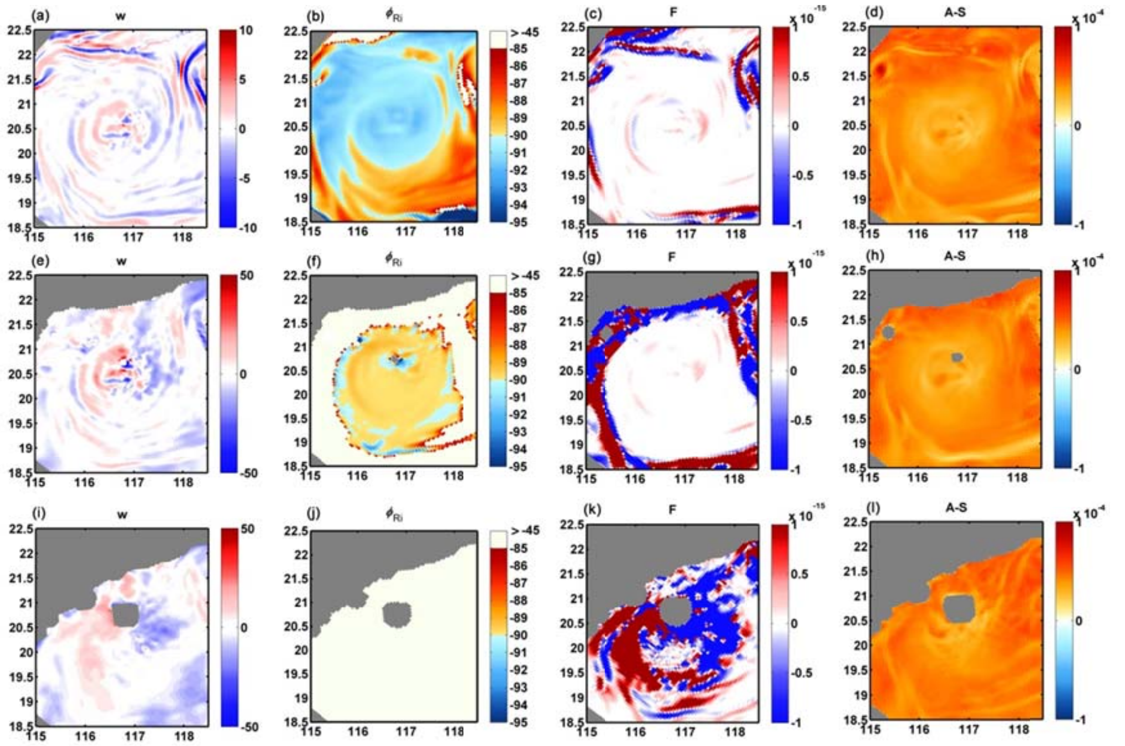

Figure SI 3: Vertical velocity (a,e,i, unit: m day^{-1}), ϕ_{Ri} (b,f,j, unit: degree), front tendency (c,g,k, unit: kg^2 m^{-8} s^{-1}) and *A*-*S* (d,h,l, unit: s^{-1}) of the MR simulations at 5 m (top), 100 m (middle) and 250 m (bottom). The contours on the *A*-*S* map are their zero-crossing lines. The figure was made using MATLAB R2010b (http://www.mathworks.com).

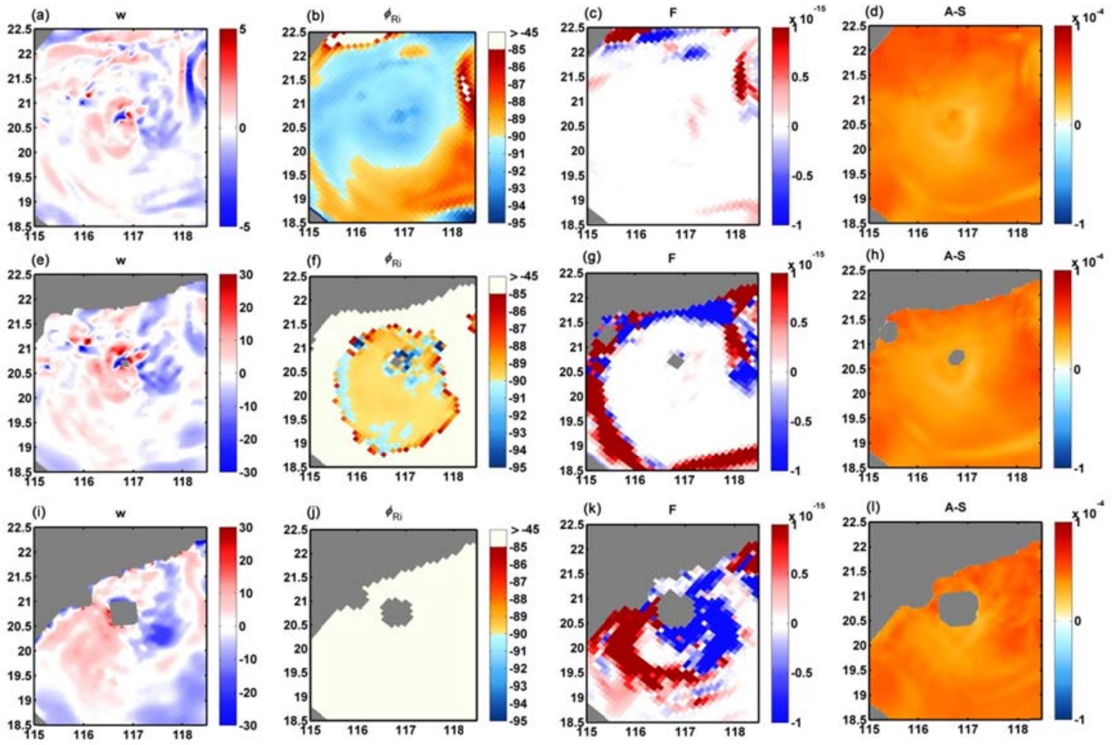

Figure SI 4: Vertical velocity (a,e,i, unit: m day^{-1}), ϕ_{Ri} (b,f,j, unit: degree), front tendency (c,g,k, unit: kg^2 m^{-8} s^{-1}) and *A*-*S* (d,h,l, unit: s^{-1}) of the MP simulations at 5 m (top), 100 m (middle) and 250 m (bottom). The contours on the *A*-*S* map are their zero-crossing lines. The figure was made using MATLAB R2010b (http://www.mathworks.com).

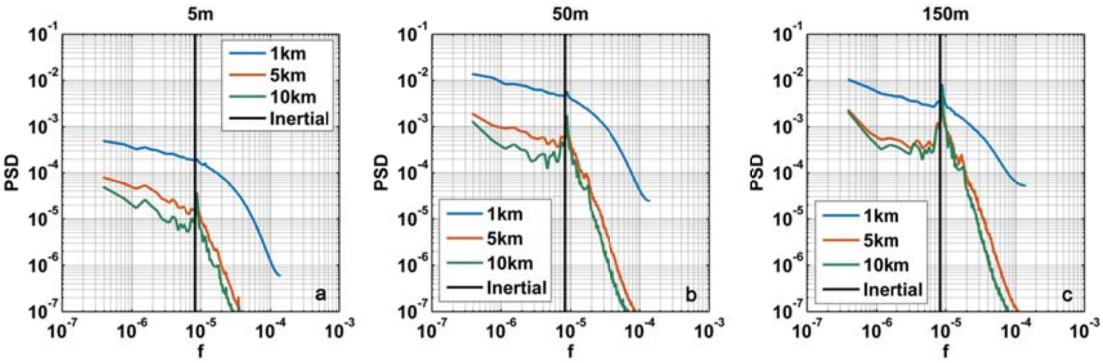

Figure SI 5: Horizontally-averaged vertical velocity spectra at 5 m, 50 m and 150 m depth in the SP (blue), MR (red) and MP (green) simulations. The black line indicates the inertial frequency. The figure was made using MATLAB R2010b (http://www.mathworks.com).

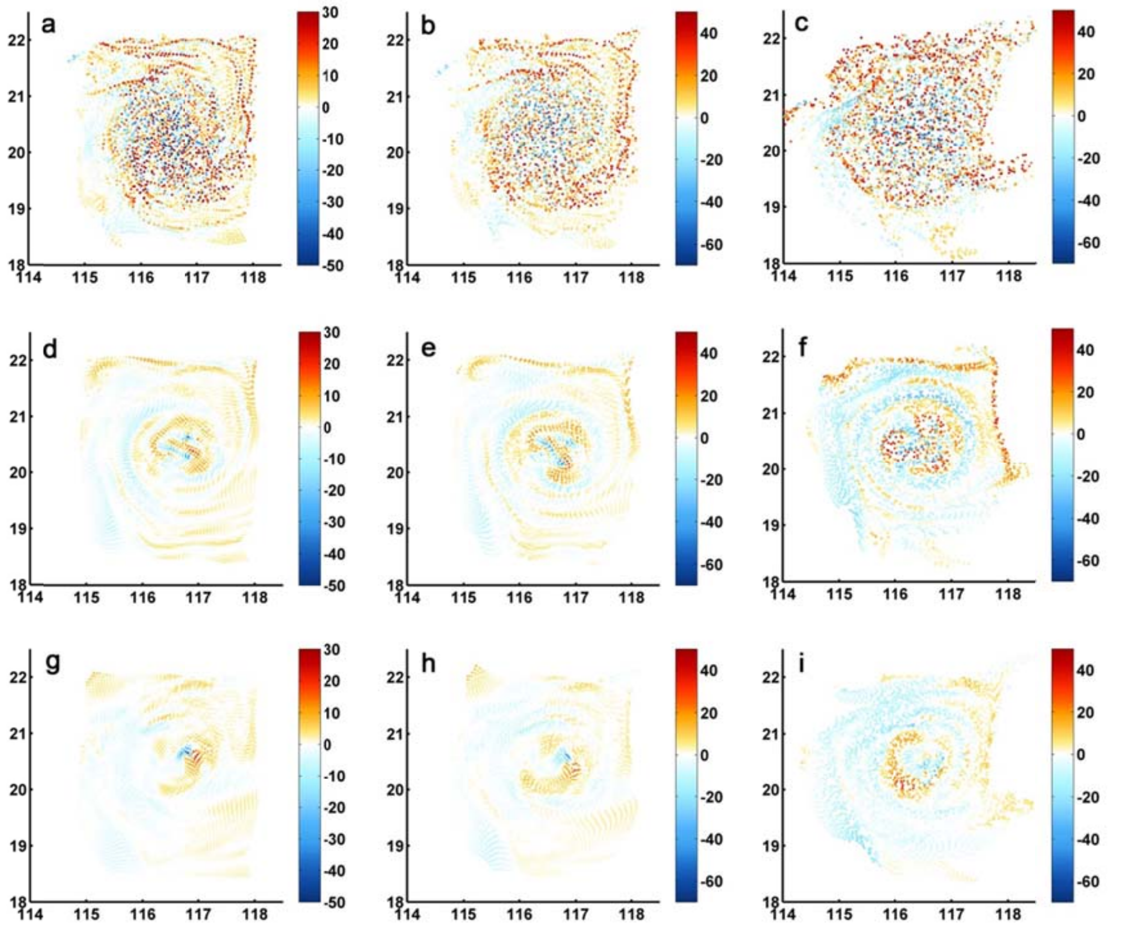

Figure SI 6: Three-dimensional distribution of particles initially deployed at 50 m after 1 day (a,d,g), 2 days (b,e,h) and 5 days (c,f,i) in the SP (top), MR (middle) and MP (bottom) simulations. Color shading indicates the particle displacement, positive for upward and negative for downward. The figure was made using MATLAB R2010b (http://www.mathworks.com).

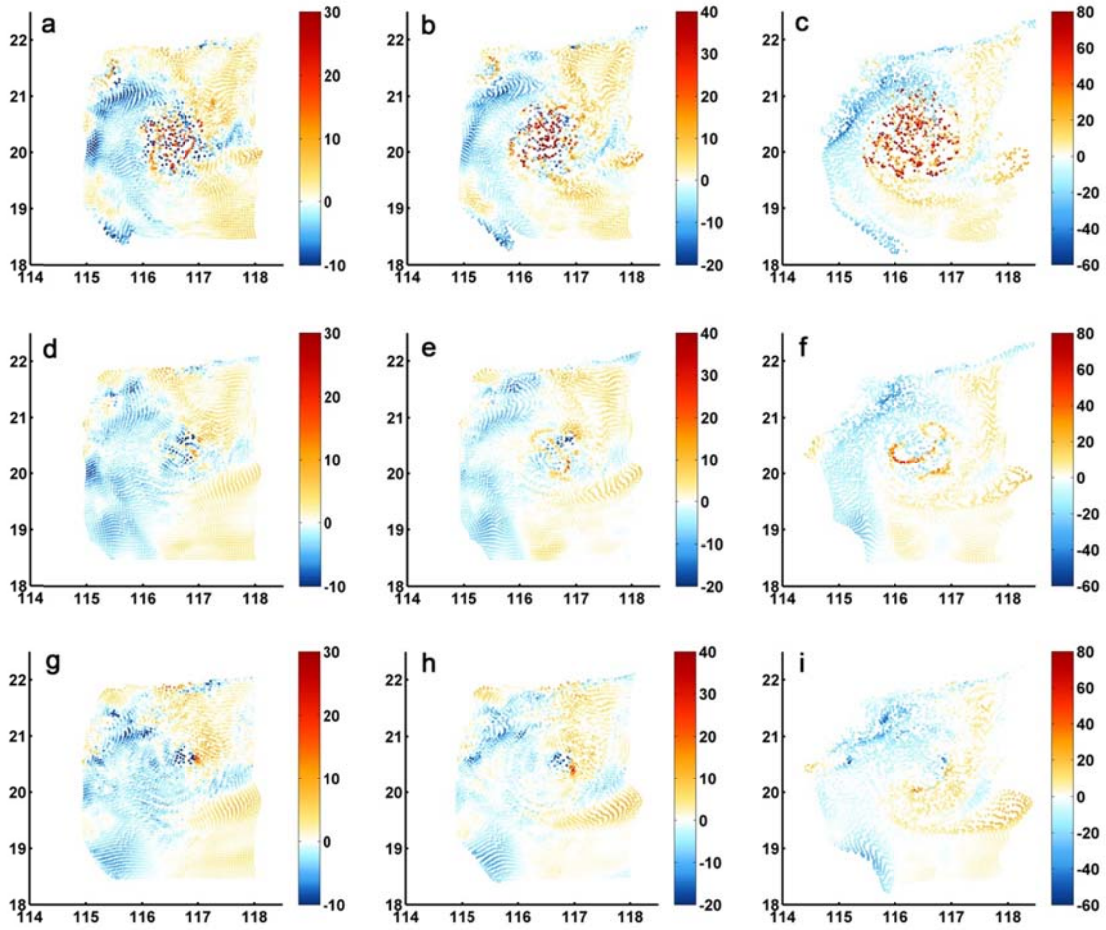

Figure SI 7: Three-dimensional distribution of particles initially deployed at 100 m after 1 day (a,d,g), 2 days (b,e,h) and 5 days (c,f,i) in the SP (top), MR (middle) and MP (bottom) simulations. Color shading indicates the particle displacement, positive for upward and negative for downward. The figure was made using MATLAB R2010b (http://www.mathworks.com).

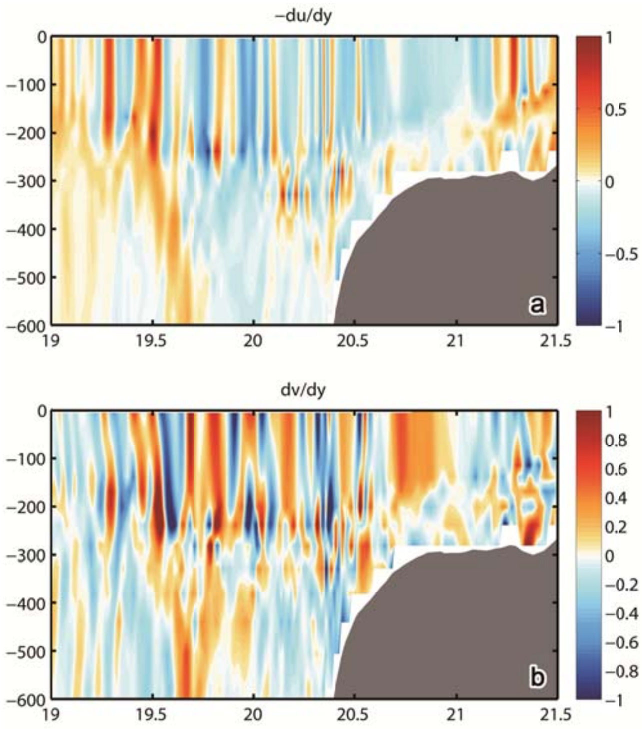

Figure SI 7: Simplified vorticity (a) and divergence (b) with no azimuthal variation in the SP model. The figure was made using MATLAB R2010b (http://www.mathworks.com).
